# Intraoperative recognition of persistent left superior vena cava during right internal jugular central line placement in mitral valve replacement: a case report

**DOI:** 10.1186/s13256-025-05698-x

**Published:** 2025-12-06

**Authors:** John Choi, Michelle Chen

**Affiliations:** Department of Anesthesiology, Mount Sinai West and Morningside Hospitals, 1000 10Th Ave, New York, NY 10019 USA

**Keywords:** Persistent left superior vena cava, Central venous catheterization, Transesophageal echocardiography, Mitral valve repair, Coronary sinus, Congenital venous anomaly

## Abstract

**Background:**

Persistent left superior vena cava is a rare congenital venous anomaly, present in approximately 0.3–0.5% of the general population and in up to 10% of patients with congenital heart disease. Although typically asymptomatic and often discovered incidentally, persistent left superior vena cava may complicate central venous catheter placement and transesophageal echocardiography confirmation during cardiac surgery. Unrecognized persistent left superior vena cava can lead to misinterpretation of guidewire position, unnecessary catheter manipulation, and procedural delays.

**Case presentation:**

A 65-year-old white woman (body mass index 26 kg/m^2^; American Society of Anesthesiologists physical status III) with severe degenerative mitral regurgitation, hypertension, and well-controlled type 2 diabetes mellitus presented for elective mitral valve repair. After uneventful induction of general anesthesia, a right internal jugular central venous catheter was placed under ultrasound guidance without resistance, arrhythmia, or abnormal waveform. Intraoperative transesophageal echocardiography—using standard bicaval, midesophageal four-chamber, and midesophageal long-axis views—failed to visualize the guidewire or catheter tip within the superior vena cava or right atrium despite correct placement technique and a normal central venous pressure waveform. Given stable hemodynamics and appropriate venous return, the catheter was secured in place, and mitral valve repair proceeded without incident. Postoperative contrast-enhanced computed tomography identified a persistent left superior vena cava draining into an enlarged coronary sinus, accounting for the atypical guidewire trajectory. The central line remained functional throughout, and the patient was extubated on postoperative day 1. She experienced an uncomplicated recovery and was discharged home on postoperative day 4.

**Conclusion:**

When transesophageal echocardiography fails to confirm central venous catheter position despite proper technique, rare anatomical variants such as persistent left superior vena cava should be considered. Employing targeted transesophageal echocardiography interrogation of the coronary sinus and adjunctive imaging modalities can facilitate prompt recognition. Awareness of persistent left superior vena cava prevents unnecessary catheter manipulation, reduces procedural delays, and enhances patient safety during cardiac surgery.

## Background

Persistent left superior vena cava (PLSVC) is a rare congenital venous anomaly resulting from failure of the left anterior cardinal vein to regress during embryogenesis. It occurs in approximately 0.3–0.5% of the general population and in up to 10% of individuals with congenital heart disease [1, 2]. In most cases, the PLSVC drains into the coronary sinus, preserving normal right-sided hemodynamics; however, a minority of cases may have alternate drainage pathways [4].

Although often asymptomatic and discovered incidentally, PLSVC can have significant procedural implications. During central venous catheter (CVC) insertion, the anomalous venous pathway may divert guidewires or catheters away from the expected course into the right superior vena cava and right atrium, leading to confusion regarding line position [5]. In the cardiac operating room, transesophageal echocardiography (TEE) is routinely used for real-time confirmation of CVC placement; standard bicaval and midesophageal views visualize the guidewire within the superior vena cava or at the superior vena cava–right atrium (SVC–RA) junction [3]. However, PLSVC draining into an enlarged coronary sinus may not be apparent unless specific interrogation of that structure is performed, limiting the sensitivity of standard TEE windows [9].

Early recognition of PLSVC is critical to avoid unnecessary catheter manipulation, procedural delays, and potential complications. Awareness of this anomaly and incorporation of targeted imaging—such as dedicated coronary sinus views on TEE or adjunctive modalities such as contrast-enhanced computed tomography—can facilitate prompt diagnosis and safe management in cardiac surgical patients.

## Case presentation

A 65-year-old white woman (body mass index 26 kg/m^2^; American Society of Anesthesiologists [ASA] physical status III) with severe degenerative mitral regurgitation, hypertension, and well-controlled type 2 diabetes mellitus was scheduled for elective mitral valve repair. Preoperative transthoracic echocardiography confirmed a flail P2 segment and left ventricular ejection fraction of 60%. Routine laboratory studies and chest radiograph were unremarkable.

**Fig. 1:**
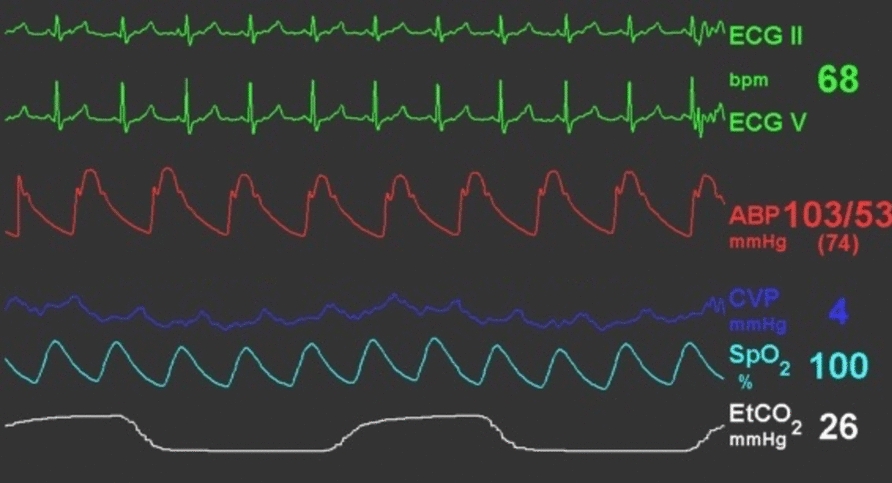
This image shows a normal central venous pressure waveform and values within normal limits

In the operating room, standard monitors were applied, and anesthesia was induced with intravenous fentanyl, etomidate, and rocuronium. After endotracheal intubation, a right internal jugular central venous catheter was inserted under real-time ultrasound guidance. The guidewire advanced smoothly without resistance, blood return was brisk, and the central venous pressure waveform was appropriate (Fig. 1).

Transesophageal echocardiography was initiated for surgical guidance and catheter confirmation. Despite imaging in standard bicaval, midesophageal four-chamber, and midesophageal long-axis views, the guidewire and catheter tip were not visualized within the superior vena cava or right atrium. Hemodynamics remained stable throughout. Given correct ultrasound-confirmed access and normal waveform, the catheter was secured, and the mitral valve repair proceeded uneventfully.

**Fig. 2:**
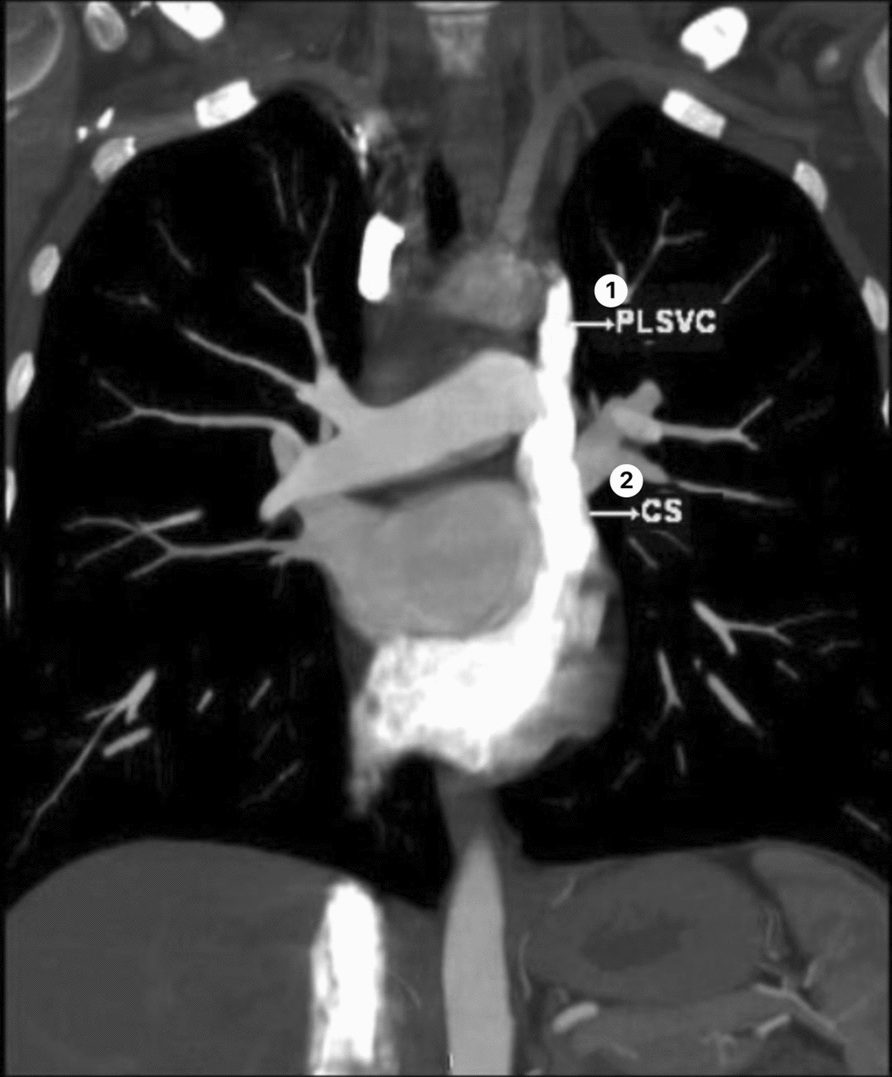
This image shows contrast-enhanced computed tomography revealing a persistent left superior vena cava draining into a dilated coronary sinus. (1) PLSVC, persistent left superior vena cava; (2) CS, coronary sinus

Postoperative contrast-enhanced computed tomography on the first postoperative day revealed a persistent left superior vena cava draining into a dilated coronary sinus (Fig. 2), explaining the aberrant guidewire trajectory. The central line functioned normally during the entire perioperative period. The patient was extubated later that day, experienced no complications, and was discharged home on postoperative day 4.

## Discussion

**Fig. 3:**
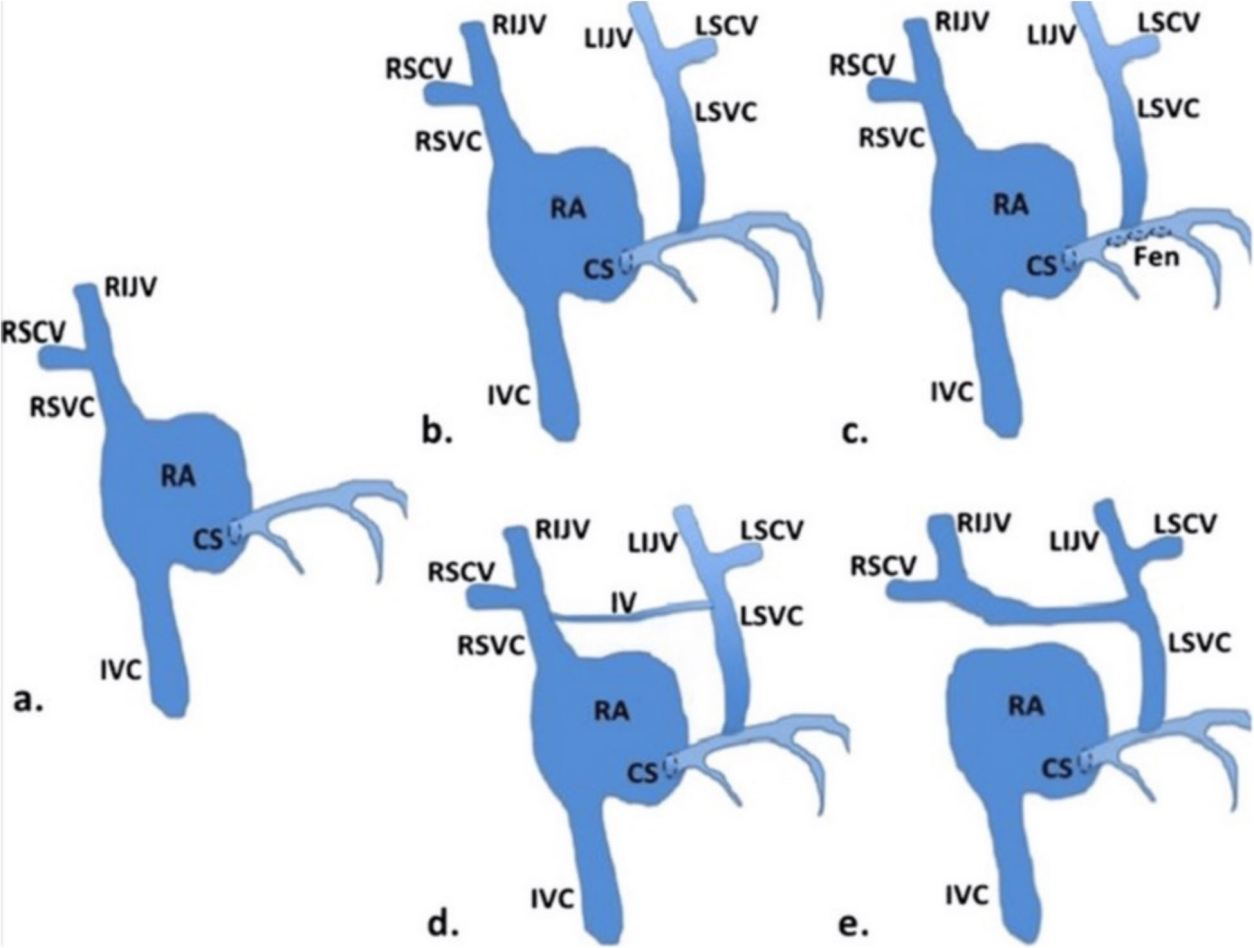
Examples of certain persistent left superior vena cava anatomic variations. (**a**) Typical venous drainage into the right atrium. (**b**) Persistent left superior vena cava and its tributaries draining into the coronary sinus. (**c**) Persistent left superior vena cava draining into the left atrium by means of an unroofed coronary sinus. (**d**) Persistent left superior vena cava draining into the coronary sinus and also connected to the right superior vena cava by an innominate vein. (**e**) Persistent left superior vena cava with an absent right superior vena cava as identified in this case report. CS, coronary sinus; Fen, fenestrations; IV, innominate vein; IVC, inferior vena cava; LIJV, left internal jugular vein; LSCV, left subclavian vein; LSVC, left superior vena cava; RA, right atrium; RIJV, right internal jugular vein; RSCV, right subclavian vein; RSVC, right superior vena cava [4] (permission to use the image was obtained from the original authors)

Persistent left superior vena cava (PLSVC) is a rare congenital venous anomaly resulting from failure of the left anterior cardinal vein to regress during embryogenesis. It occurs in approximately 0.3–0.5% of the general population and in up to 10% of patients with congenital heart disease [1, 2]. In most cases, PLSVC drains into the coronary sinus, preserving normal right-sided circulation, though alternate drainage patterns exist [2]. Variations include drainage into the left atrium in approximately 8% of cases, a double superior vena cava with or without a bridging innominate vein, and rare absence of the right SVC [4], each of which carries distinct clinical implications (Fig. 3).

Transesophageal echocardiography (TEE) is routinely used intraoperatively to confirm central venous catheter placement by visualizing the guidewire [8] within the superior vena cava or at the SVC–RA junction in bicaval and midesophageal views [7]. PLSVC diverts the catheter into the left mediastinum and coronary sinus, which standard TEE windows may not interrogate, leading to false assumptions of malposition [3]. Failure to recognize PLSVC intraoperatively can result in unnecessary catheter manipulation, procedural delays, and potential complications.

Adjunctive imaging modalities such as chest radiography and contrast-enhanced computed tomography can delineate the anomalous venous course and confirm the diagnosis [6]. An enlarged coronary sinus on TEE or atypical catheter trajectory on radiograph should prompt suspicion for PLSVC [10]. Targeted TEE interrogation of the coronary sinus using dedicated long-axis or modified bicaval views enhances detection [11].

Beyond CVC placement, PLSVC has critical implications for pacemaker and defibrillator lead placement, retrograde cardioplegia delivery, and cardiopulmonary bypass cannulation strategies [4, 9]. Rarely, PLSVC drains into the left atrium, creating a right-to-left shunt with risk of paradoxical embolism, underscoring the importance of accurate identification [12].

Our case highlights the need for a high index of suspicion when standard TEE confirmation of CVC fails despite correct technique. Incorporating multimodal imaging and targeted echocardiographic views enables prompt recognition of PLSVC, prevents unnecessary interventions, and promotes patient safety in cardiac surgery.

## Conclusion

Persistent left superior vena cava is a rare congenital venous anomaly that may unexpectedly complicate central venous access and intraoperative imaging during cardiac surgery. When standard transesophageal echocardiography views fail to confirm central venous catheter placement despite correct technique and normal catheter function, clinicians should consider variant venous anatomy. Awareness of PLSVC and its anatomic variations—including drainage into the coronary sinus or left atrium, double SVC, and absent right SVC—allows for targeted TEE interrogation and use of adjunctive imaging modalities such as contrast-enhanced computed tomography. Early recognition of PLSVC prevents unnecessary catheter manipulation, reduces procedural delays, and enhances patient safety in mitral valve repair and other cardiac procedures.

## Data Availability

All relevant data supporting the findings of this case report are included within the article.

## Abbreviations

ASA: American Society of Anesthesiologists
BMI: Body mass index
CVP: Central venous pressure
CVC: Central venous catheter
CT: Computed tomography
TEE: Transesophageal echocardiography
PLSVC: Persistent left superior vena cava
RA: Right atrium
SVC: Superior vena cava
RIJ: Right internal jugular

## References

[1] Goyal SK, Punnam SR, Verma G, Ruberg FL. Persistent left superior vena cava: a case report and review of literature. Cardiovasc Ultrasound. 2008. 10.1186/1476-7120-6-50. 18847480 10.1186/1476-7120-6-50 PMC2576163

[2] Gonzalez-Juanatey C, Testa A, Vidan J, Izquierdo R, Garcia-Castelo A, Daniel C, Armesto V. Persistent left superior vena cava draining into the coronary sinus: report of 10 cases and literature review. Clin Cardiol. 2004. 10.1002/clc.4960270909. 15471164 10.1002/clc.4960270909 PMC6654321

[3] Ender J, Erdoes G, Krohmer E, Olthoff D, Mukherjee C. Transesophageal echocardiography for verification of the position of the electrocardiographically-placed central venous catheter. J Cardiothorac Vasc Anesth. 2009. 10.1053/j.jvca.2008.12.003. 19217801 10.1053/j.jvca.2008.12.003

[4] Rizkallah J, Burgess J, Kuriachan V. Absent right and persistent left superior vena cava: troubleshooting during a challenging pacemaker implant: a case report. BMC Res Notes. 2014. 10.1186/1756-0500-7-462. 25047923 10.1186/1756-0500-7-462 PMC4112616

[5] Yoshimura M, Nakanishi T, Sakamoto S, Toriumi T. Confirmation of optimal guidewire length for central venous catheter placement using transesophageal echocardiography. J Clin Anesth. 2016. 10.1016/j.jclinane.2016.07.032. 27871596 10.1016/j.jclinane.2016.07.032

[6] Laurenzi L, Natoli S, Pelagalli L, Marcelli ME, Abbattista D, Carpanese L, Arcuri E. Long-term central venous catheterization via persistent left superior vena cava: a case report. Support Care Cancer. 2003. 10.1007/s00520-002-0421-9. 12618930 10.1007/s00520-002-0421-9

[7] Jeon Y, Ryu HG, Yoon SZ, Kim JH, Bahk JH. Transesophageal echocardiographic evaluation of ECG-guided central venous catheter placement. Can J Anaesth. 2006. 10.1007/BF03022525. 16987851 10.1007/BF03022525

[8] Andropoulos DB, Stayer SA, Bent ST, Campos CJ, Bezold LI, Alvarez M, Fraser CD. A controlled study of transesophageal echocardiography to guide central venous catheter placement in congenital heart surgery patients. Anesth Analg. 1999. 10.1097/00000539-199907000-00012. 10389780 10.1097/00000539-199907000-00012

[9] Pálinkás A, Nagy E, Forster T, Morvai Z, Nagy E, Varga A. A case of absent right and persistent left superior vena cava. Cardiovasc Ultrasound. 2006. 10.1186/1476-7120-4-6. 16438718 10.1186/1476-7120-4-6 PMC1382278

[10] Kurtoglu E, Cakin O, Akcay S, Akturk E, Korkmaz H. Persistent left superior vena cava draining into the coronary sinus: a case report. Cardiol Res. 2011. 10.4021/cr85w. 28357015 10.4021/cr85w PMC5358287

[11] Kurusz M, Girouard MK, Brown PS Jr. Coronary sinus rupture with retrograde cardioplegia. Perfusion. 2002. 10.1191/0267659102pf534cr. 11817534 10.1191/0267659102pf534cr

[12] Sharma S, Devine W, Anderson RH, Zuberbühler JR. The determination of atrial arrangement by examination of appendage morphology in 1842 heart specimens. Br Heart J. 1988. 10.1136/hrt.60.3.227. 3179139 10.1136/hrt.60.3.227 PMC1216558

